# Foldify: Web Application for Protein Structure Prediction

**DOI:** 10.1021/acs.jcim.6c01154

**Published:** 2026-06-26

**Authors:** Romana Ďuráčiová, Michaela Capandová, Karel Berka, Radka Svobodová, Terézia Slanináková, Kristián Kováč, Matej Antol, Lukáš Hejtmánek

**Affiliations:** † Institute of Computer Science, Masaryk University, Brno 628 00, Czech Republic; ‡ Department of Physical Chemistry, Faculty of Science, Palacký University Olomouc, 17. Listopadu 12, Olomouc 779 00, Czech Republic; § CEITEC, Masaryk University, Brno 62500, Czech Republic

## Abstract

Protein structure prediction models released in recent years have presented tectonic changes in the field of structural biology. However, their potential has not yet been harnessed to its fullest due to their demands on hardware and technical expertise required for their usage. In this paper, we present Foldify, which makes prediction models accessible, integrating AlphaFold 3, AlphaFold 2, ColabFold, OmegaFold, and ESMFold into a single user-friendly, easy-to-use graphical interface, and ensures their stable operation within a scalable high-performance computing environment. Foldify accepts protein sequences, submitted through a web-based graphical interface as input, and allows executing multiple prediction models on the same protein sequence. The predicted protein structures can be directly visualized online through Mol* Viewer or can be downloaded from the website. Furthermore, the multiresult comparison mode allows visualization of multiple predicted structures in a single Mol* window, accompanied by qualitative metrics of the models’ prediction similarity. The Foldify application is freely available at https://foldify-open.cloud.e-infra.cz/ with no login required.

## Graphical abstract

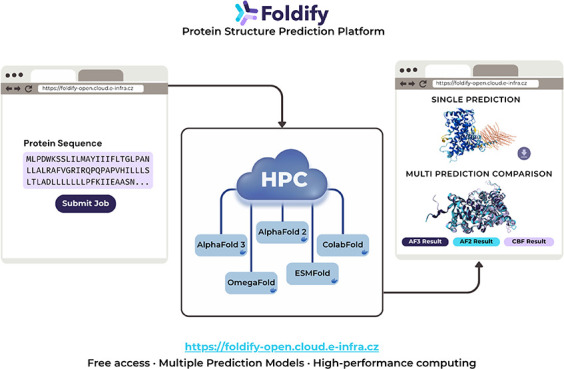

## Introduction

Predicting accurate protein structures from amino acid sequences has long been a challenge in biochemistry. Traditional experimental methods for structure determination are costly and time-consuming. However, recent advancements in artificial intelligence and deep learning have enabled significant progress in computational protein structure prediction, with AlphaFold 2 ^1^ pioneering the demonstration that protein structures can be predicted with accuracy sufficient for many practical applications, subsequently influencing a whole class of prediction methods.^2–4^ This growing diversity of prediction tools offers practical advantages, as different methods vary in speed and performance on specific types of protein sequences, allowing researchers to select and test prediction tools that best match their research objectives.^5^

Despite these advancements, the accessibility of protein prediction tools has been hindered by their computational intensity, warranting HPC infrastructure and the advanced technical expertise needed for their installation and operation. Such constraints create barriers for the scientists and researchers who would benefit from the software-driven prediction yet lack the technical knowledge and computational infrastructure.

This challenge has motivated the development of three web platforms for protein structure prediction: AlphaFold Server,^6^ Neurosnap,^7^ and ProteinIQ.^8^ These services are, however, limited in the extent of the offered functionality. AlphaFold Server needs connection to a Google account and only provides the AlphaFold 3 model.^9^ Neurosnap, while enabling access to multiple models (AlphaFold 3, AlphaFold 2, ESMFold, OmegaFold, etc.), is, similarly to ProteinIQ, primarily a paid service. Neurosnap’s free tier is exhausted after approximately four predictions of a medium-sized protein such as porin,^10^ while ProteinIQ’s initial 100 complimentary credits do not cover even a single prediction of the same protein (using the AlphaFold 2 prediction tool).

To reduce the technical complexity and provide a free and easy interface for protein prediction, we developed Foldify. Foldify is a web application that centralizes access to the popular protein prediction tools (namely, AlphaFold 3,^9^ AlphaFold 2,^1^ ColabFold,^2^ OmegaFold,^4^ and ESMFold^3^) within a single platform. It allows running multiple simultaneous predictions and integrates an analysis portal for the output with rich visualization, confidence (pLDDT), and structural alignment metrics (RMSD, TM-Score^11^). The tool is deployed on a cloud computing HPC environment of e-INFRA CZ^12^ and is free to use at https://foldify-open.cloud.e-infra.cz.

In its current deployment, Foldify allows users to predict protein structures of up to 600 amino acids in length. This limit covers most typical use cases, as proteins that are typically the focus of structural studies in humans and other eukaryotes are around a few hundred amino acids in length, with most functional domains falling well within this range.^13,14^ We have also made the code publicly available to facilitate the deployment of Foldify on any high-performance hardware.

## Implementation

### Interface

The Foldify user interface consists of three main pages: the Dashboard, the Submission Form, and the Result Page (see Figure 1). The Dashboard (Figure 1A) shows an overview of the available folding tools, a summary of running and completed computations, and a results section with three example proteins that allows users to explore the results page without waiting for job completion. Users begin their computation by selecting a prediction model from the Dashboard. Apart from individual models, Foldify allows running predictions using multiple models (“MultiFold” option). Users are then redirected to the Submission Form page (Figure 1B), which requires an email address, used for notification about job’s completion, and the protein sequence. After the job is submitted, its status can be monitored from the “Running & Completed Computations” section of the Dashboard (Figure 1C), with detailed logs accessible via the “View Details” link. When the computation is finished and the result is ready for visualization, the user receives an email notification containing a direct URL to the corresponding Result Page within the Foldify interface. The results remain accessible via the Dashboard’s Running & Completed Computations table for 7 days. By default, all results are marked as “Public” and get a persistent URL with a minimum retention period of 30 days. All generated data are available for local download. These include the predicted conformations in CIF or PDB format, global confidence metrics, predicted aligned error matrices where applicable, model input features and metadata, ranking scores or model ranking information, and inference logs. However, the exact files and formats vary by the selected folding method. These outputs can be further utilized in downstream analyses, including molecular docking, binding-site identification, structural similarity searches, protein stability assessment, or molecular dynamics simulations. In the case of the “MultiFold” option, once the computations have finished, the user can select a reference protein and one or more models for comparison. The multicomparison result page (Figure 1D) presents both graphical visualizations and quantitative comparison metrics of the models. Persistent public access (30 days minimum) requires that all models in the comparison are marked as “Public”.

**Figure 1:**
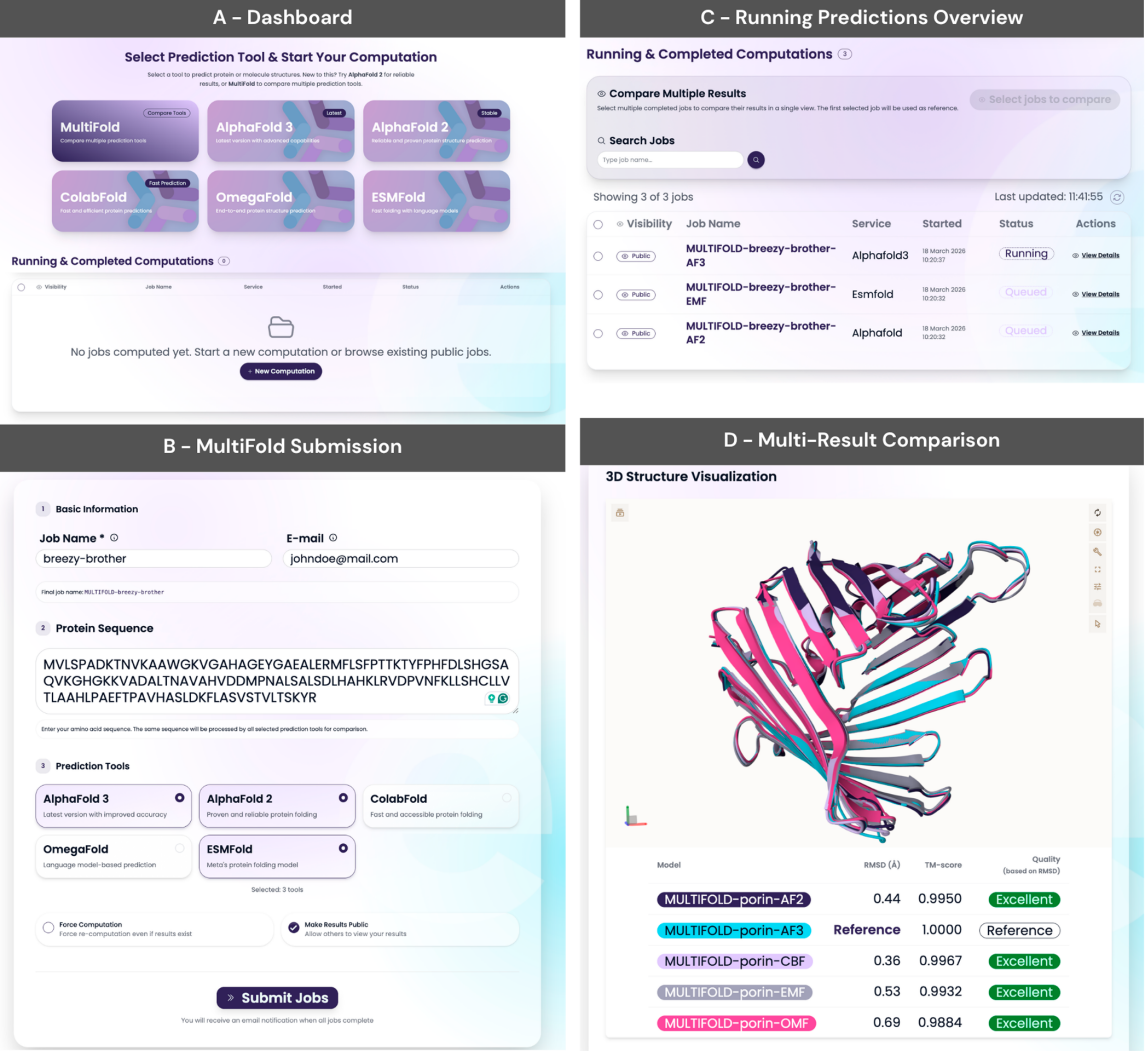
Overview of the Foldify web interface. (A) Dashboard displaying available prediction tools and the job monitoring panel. (B) MultiFold submission interface enabling structure prediction with user-specified parameters and settings. (C) Job management table showing computation status for submitted predictions. (D) Multiresult comparison interface presenting structural superpositions (top) and quantitative comparison metrics (bottom), including RMSD (Å) and TM-scores, for comparative assessment of model predictions against a designated reference structure.

### Architecture

The architecture of the application, as illustrated in Figure 2, consists of two main components: web application frontend and API server backend, both containerized using Docker and deployed within a Kubernetes environment. The API server is responsible for running the prediction jobs, managing and storing both input and output data in persistent storage, as well as validating and formatting the input.

**Figure 2:**
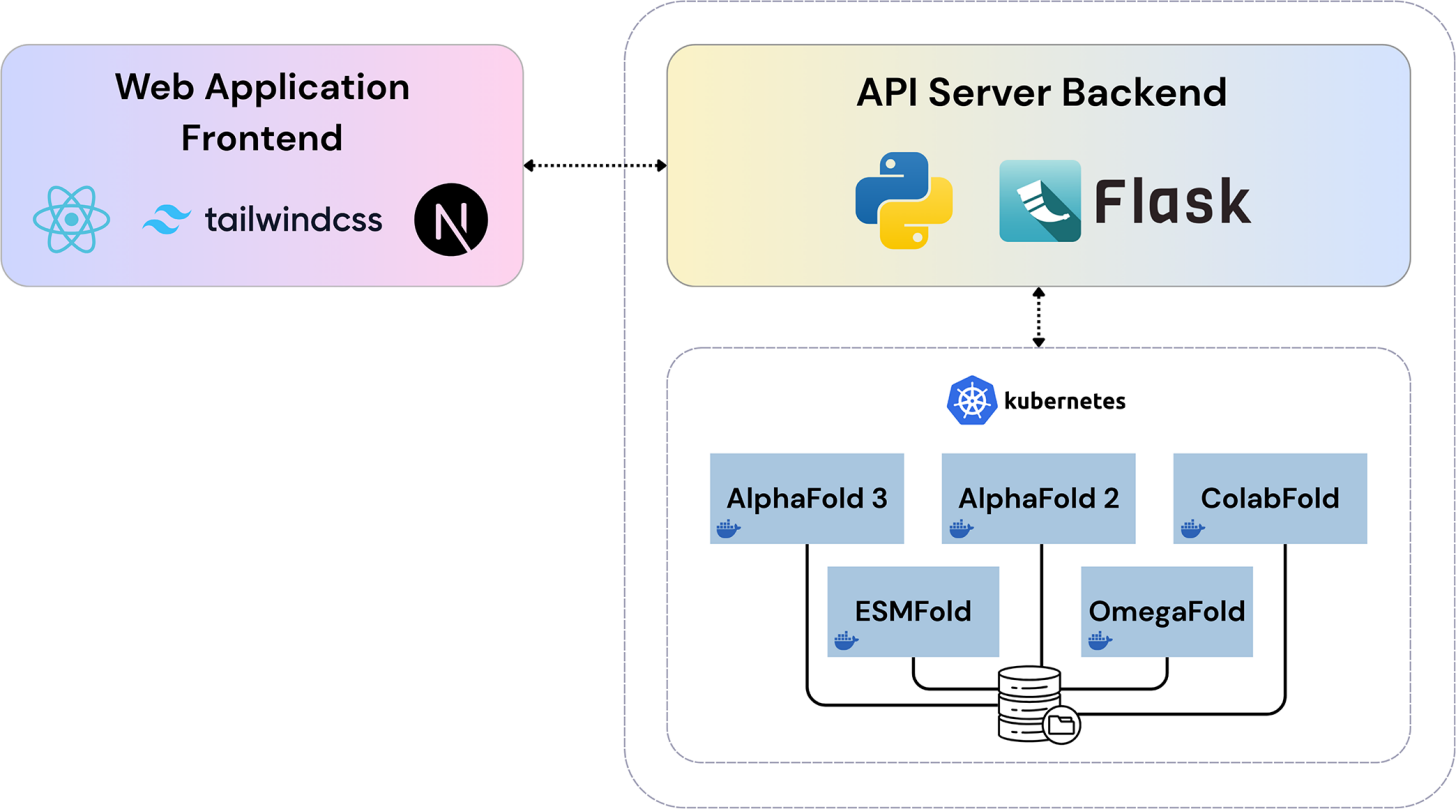
System architecture of the Foldify platform. The platform implements a containerized, service-oriented architecture consisting of a Next.js frontend (left) and a Python API server (upper right) that manages computations within the Kubernetes cluster (lower right). User requests are processed through API calls to the backend server, which orchestrates job execution across Docker-containerized prediction tools (AlphaFold 3, AlphaFold 2, ColabFold, OmegaFold, ESMFold) with access to shared storage for input/output data persistence.

The frontend accessible via a web browser allows users to submit computational requests and analyze prediction results via visualizations, multiresult comparisons, and the display of computed quantitative metrics.

The Kubernetes cluster coordinates computations and provides the resources to spawn containers with submitted predictions. The images of the prediction tools are preprepared and ready to run the predictions on demand. Computed data are then stored in the persistent volume, ready for retrieval by the frontend.

### Used Technologies

To ensure broad accessibility, Foldify is publicly available as a ready-to-use web service hosted on the e-INFRA CZ infrastructure. Moreover, the complete source code is available on GitHub,^15^ enabling the deployment of the application within any computational environment (preferably Kubernetes infrastructure), with the necessary resources available.

The frontend was developed in React using the Next.js framework^16^ and TypeScript.^17^ The responsive UI design was achieved using the Tailwind CSS library^18^ and components from the daisyUI library.^19^ The backend was built using Python and Flask^20^ leveraging the BioPython library^21^ for processing protein data.

### Prediction Model Optimization

All five prediction models (AlphaFold 3, AlphaFold 2, ColabFold, OmegaFold, and ESMFold) were downloaded from their respective GitHub repositories and prepared as individual Docker containers. To avoid performance bottlenecks, specifically for the AlphaFold 3 model, we redesigned the pipeline into a Kubernetes-native architecture. This included splitting the computation into two phases: CPU-exclusive (building multiple sequence alignments with the HMMER package^22^) and GPU-accelerated (running the structure prediction itself).

### Limitations

Protein structure prediction is computationally intensive; consequently, processing times may vary depending on the server load and resource availability. To ensure equitable resource distribution, the system limits each user to five concurrently running jobs, including additional submissions that are queued until processing capacity becomes available.

Due to limited storage and generally large outputs, Foldify guarantees output persistence for a maximum of 30 days. After this period, the results are no longer available. For persistence, we encourage the users to download their results.

## Results and Discussion

We have deployed and monitored Foldify for the past year on the e-INFRA CZ research infrastructure to ensure stability and gather community feedback. The platform was, and continues to be, in active use by the Czech research community across multiple academic institutions. Figure 3 shows the gathered usage statistics of the various prediction models. Three models, AlphaFold 2, AlphaFold 3, and ColabFold, account for over 97% of Foldify’s usage. This is in line with the literature, as AlphaFold 2 and 3 models are known for high predictive performance and strong visibility in the community^23^ and ColabFold is known for its favorable balance between reliability and computational efficiency. These results support the rationale for offering multiple prediction models, as it enables users to select the most suitable tool based on their specific needs.

**Figure 3:**
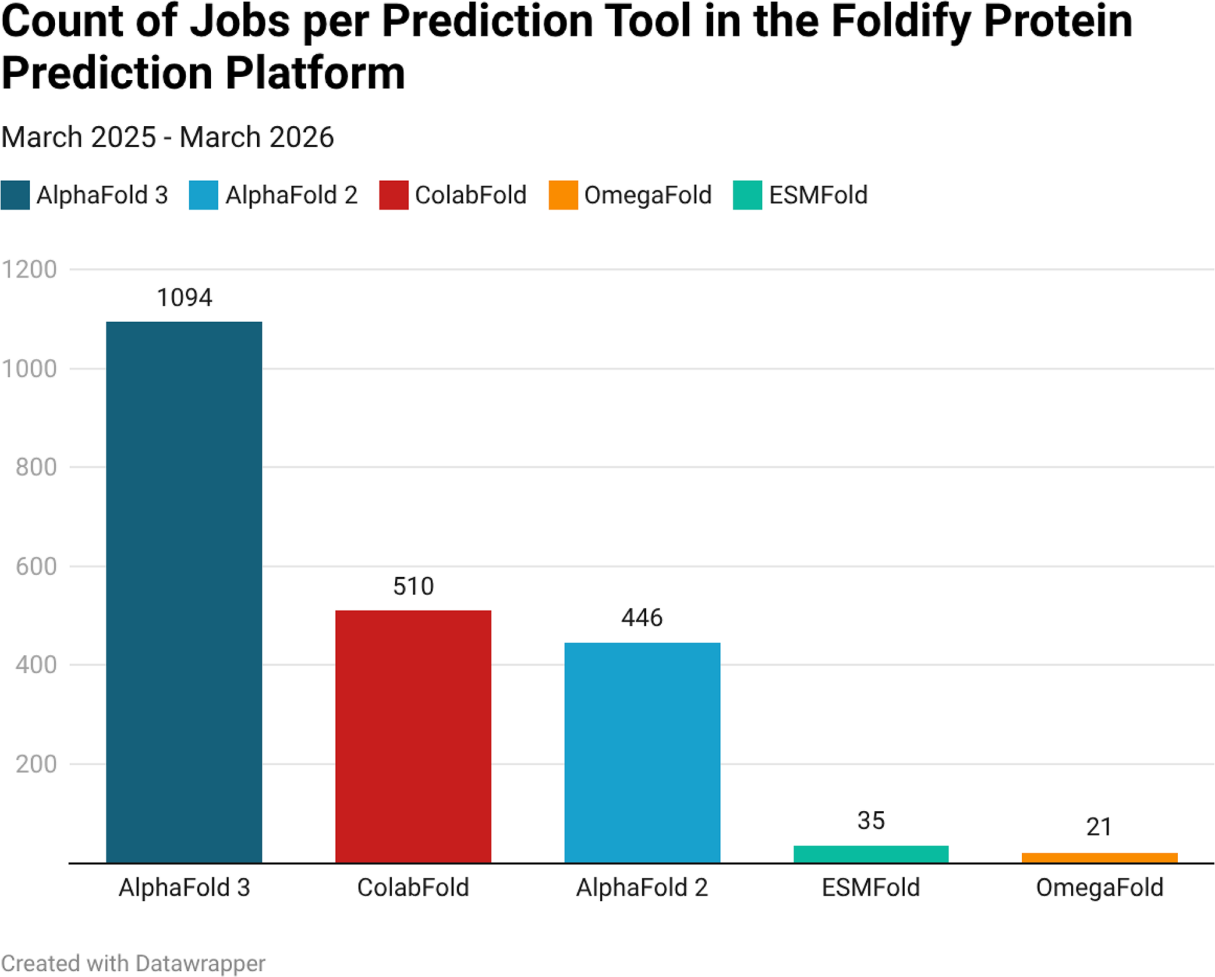
Distribution of prediction tool usage on the Foldify platform. The platform processed 2 106 total jobs during the whole pilot year (March 2025–March 2026), with AlphaFold 3 being the most utilized tool with 1 094 computed jobs. ColabFold and AlphaFold 2 showed moderate usage (510 and 446 jobs), while ESMFold and OmegaFold were rarely selected (35 and 21 jobs).

To illustrate the practical applications of Foldify, we present three representative use cases, carefully selected with biological relevance in mind. Specifically, we demonstrate comparative structural analysis on Porin, fatty acid addition on membrane protein cytochrome P450, and enzymatic features on dipeptidyl peptidase, highlighting the diverse capabilities of Foldify across different protein types.

### Use Case I: Structurally Conserved Protein—Porin

The first use case focuses on the porin protein VDAC2 (voltage-dependent anion-selective channel protein 2), which belongs to the widespread and well-known family of VDACs (voltage-dependent anion-selective channels). These proteins are mitochondrial porins, forming an ion channel located in the outer mitochondrial membrane.^24^ This channel serves as a general diffusion pore for small hydrophilic molecules.^25^ The channel has an open conformation at low or zero membrane potential and a closed conformation at potentials above 30–40 mV.^26^ In general, VDAC2 shares biological functions with other VDAC isoforms. Moreover, it plays an important role in mammalian cardiomyocytes, where it promotes the mitochondrial transport of calcium ions to power cardiac contractions.

The porin protein VDAC2 (UniProt ID P45880) forms a compact yet flexible β barrel, composed of 19 strands, including one internal helix. Its structure is strongly conserved, making it a good benchmark protein for comparing all five structure-predicting tools included in Foldify. We expected that all five predictors would provide the same 3D structure. Foldify fully confirmed this hypothesis (see Figure 4): The TM-score of all the tools compared to AlphaFold3 is >0.98, and the RMSD is <0.7 Å, which corresponds to an essentially identical fold. The closest agreement with the reference was observed for ColabFold, followed by AlphaFold2 and ESMFold, while OmegaFold still remained within an excellent similarity regime by RMSD. These results indicate that for a compact β barrel membrane protein such as VDAC2, Foldify enables straightforward cross model comparison and confirms that current predictors converge strongly on the same global topology, supporting the use of any of these models as a reliable starting point for membrane aware analyses and for downstream simulation setup where correct barrel placement and loop topology are critical. This use case also demonstrates that all predictors are integrated correctly into Foldify. For visualization of the predicted models of the porin via the Foldify platform, visit the following link:

**Figure 4:**
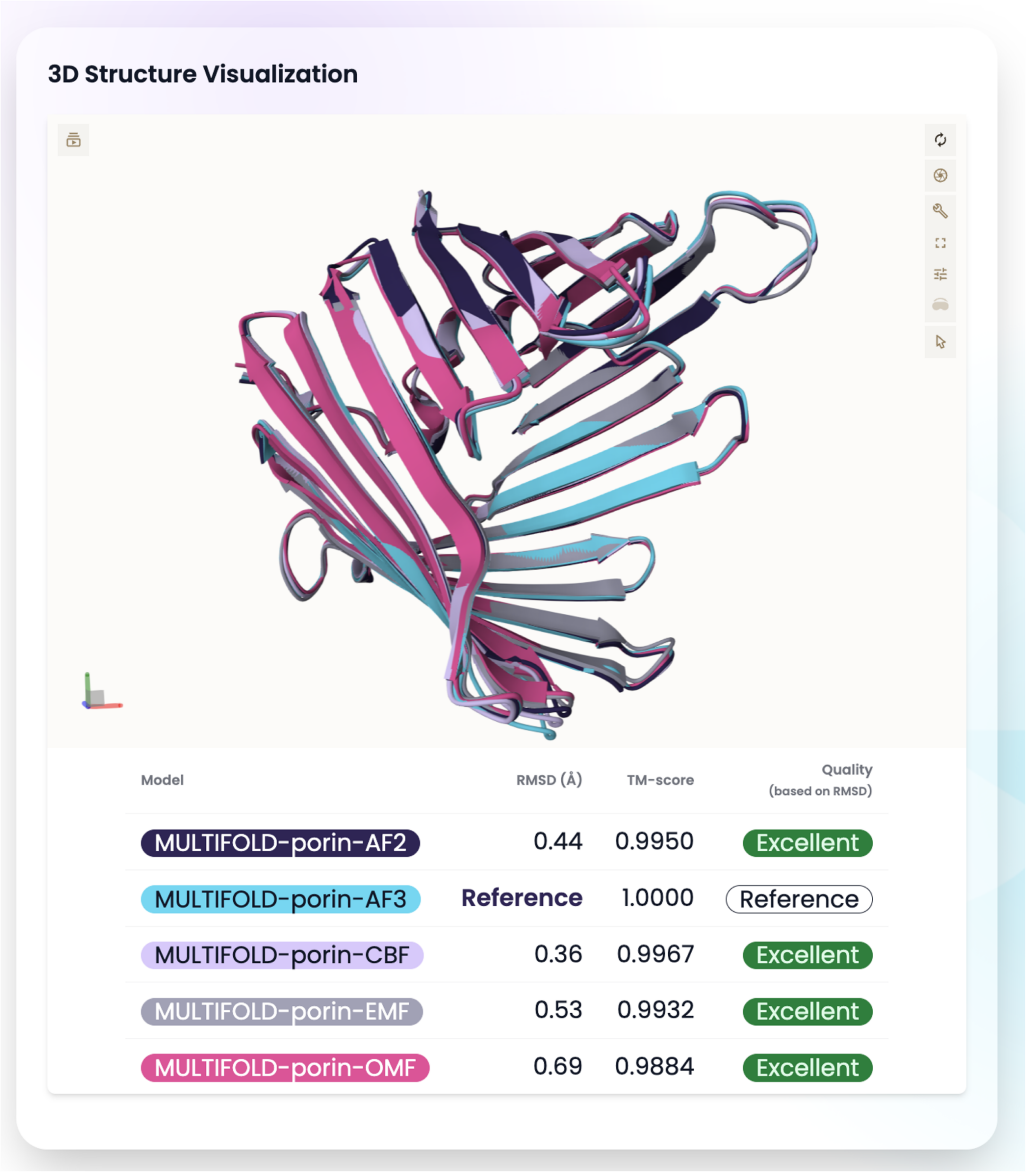
Multiresult comparison analysis of porin protein structure predictions. Porin protein input sequence was obtained from the AlphaFoldDB entry with UniProt ID P45880-2. (Upper) Superimposition of 3D structural models generated by AlphaFold 3 (reference; cyan), AlphaFold 2 (dark purple), ColabFold (violet), ESMFold (gray), and OmegaFold (pink), displayed in the integrated Mol* Viewer. (Lower) Quantitative alignment metrics relative to the AlphaFold 3 reference structure, showing RMSD values (in Å), TM-scores, and quality classifications for each prediction method. All comparison models achieved “Excellent” ratings.

https://foldify-open.cloud.e-infra.cz/result/multi/MULTIFOLD-porin-AF3_MULTIFOLD-porin-EMF_MULTIFOLD-porin-CBF_MULTIFOLD-porin-OMF_MULTIFOLD-porin-AF2.

### Use Case II: Membrane Protein

The second use case illustrates Foldify’s application on a widely studied membrane protein, cytochrome P450, with the addition of fatty acids to the model further enhancing the representation of membrane-associated features. Cytochrome P450 2C9 (CYP2C9) is one of the major drug-metabolizing proteins in the human liver, metabolizing a large range of drugs,^27^ e.g., weakly acidic NSAIDs such as ibuprofen. CYP2C9 is localized on the edge of the membrane of the endoplasmic reticulum, to which it is attached via its N-terminal anchor. Its numerous experimental crystal structures lack an N-terminal anchor, as the crystallization procedure required only a solubilized domain. The first atomistic structures of CYP2C9 on membranes were thus obtained from molecular dynamics (MD) simulations.^28,29^ MD simulations have shown that the N-terminal transmembrane anchor is not the only structure in contact with the membrane. A so-called FG-loop is also embedded in the membrane and plays an important mechanistic role, allowing lipophilic substrates to access the heme-containing active site directly from the membrane. It has been later shown that such an organization is common among all other major cytochrome P450 enzymes.^30^ Upon the release of the AlphaFold3 server in May 2024,^6^ enabling a prediction of the protein structure, including a selected list of ligands and cofactors together, it was quickly shown that AlphaFold3 can predict the membrane position in the context of protein structure.^31^ This process, called ligand cofolding, enabled mimicking a lipid membrane by adding tens of fatty acids (AF3 enables three: PLM—saturated palmitic acid, MYR—shorter myristoyl acid, and OLA—unsaturated oleic acid). These fatty acids localize in a bilayer structure resembling the lipid membrane’s hydrophobic core. In the case of CYP enzymes, they show a bilayer-like arrangement encircling the N-terminal transmembrane anchor and touching the FG-loop. This is consistent with observations from MD simulations.^30^ Excessive addition of fatty acids may cause some to occupy the hydrophobic heme-binding site, which is natural, as CYP enzymes can also oxidize lipids. Figure 5 presents the AlphaFold3-predicted structure of Cytochrome P450 2C9 in complex with heme, visualized within the Foldify platform’s integrated Mol* Viewer.

**Figure 5:**
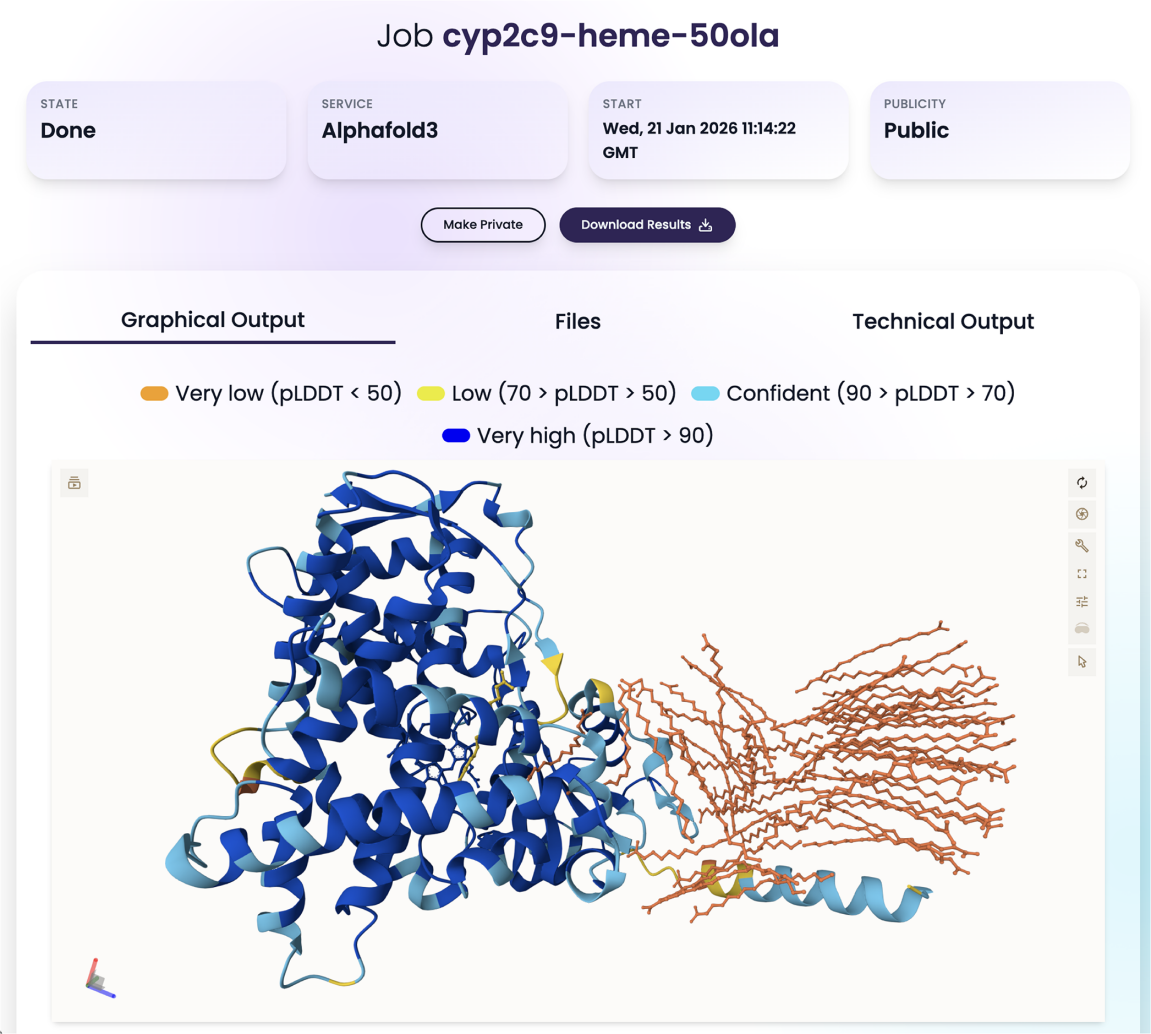
AlphaFold 3 prediction result of the Cytochrome P450 2C9. 3D structure visualization of the CYP1A2 protein with a heme cofactor, colored by predicted Local Distance Difference Test (pLDDT) confidence scores: very high confidence (dark blue, pLDDT > 90), confident (cyan, 90 > pLDDT > 70), low confidence (yellow, 70 > pLDDT > 50), and very low confidence (orange, pLDDT < 50). The predominantly blue coloration indicates high prediction confidence across most structural regions.

Foldify can be used to replicate this approach to model membrane proteins, such as all CYP enzymes mentioned in ref 30. For visualization of the precomputed models of the CYP enzymes via the Foldify platform, visit the following links:

https://foldify-open.cloud.e-infra.cz/result/cyp1a2-heme-50ola - CYP1A2.

https://foldify-open.cloud.e-infra.cz/result/cyp2a6-heme-50ola - CYP2A6.

https://foldify-open.cloud.e-infra.cz/result/cyp2c9-heme-50ola - CYP2C9.

https://foldify-open.cloud.e-infra.cz/result/cyp2d6-heme-50ola - CYP2D6.

https://foldify-open.cloud.e-infra.cz/result/cyp2e1-heme-50ola - CYP2E1.

https://foldify-open.cloud.e-infra.cz/result/cyp3a4-hem-52ola - CYP3A4.

### Use Case III: Large Multidomain Protein—Dipeptidyl Peptidase IV

Peptidases catalyze the hydrolysis of peptide bonds. Dipeptidyl peptidase IV (DPP IV)^32^ preferentially cleaves substrate peptides with Pro or Ala at a specific position of peptides. DPP IV has been isolated from bacteria, fungi, and mammals. In mammals, DPP IV is responsible for the degradation of incretins and plays a major role in glucose metabolism. DPP IV is a homodimeric enzyme, and in this use case, we focus on its N-terminal domain (UniProt ID A0A8T2CSI4), which consists of approximately 700 amino acids. Therefore, we used it as a benchmark, testing the performance of Foldify’s predictors (AlphaFold 3, AlphaFold 2, ColabFold, ESMFold, and OmegaFold) on large molecular systems.

In this use case, agreement between predictors was lower than for the porin use case, with RMSDs of 3.14 Å for ESMFold and 3.81 Å for OmegaFold, while TM scores remained high at 0.9415 and 0.9365, respectively (see Figure 6). This indicates that the predictors converge on the same global fold but differ in finer structural details, most plausibly in domain orientations and flexible surface regions, which disproportionately affect RMSD while leaving the overall topology largely unchanged. From the perspective of practical modeling, the DPP IV example highlights how Foldify can identify targets where different predictors are not strictly interchangeable at the level of atomic detail, and where selection of a particular model and subsequent refinement or validation may be important for downstream applications such as docking, interface analysis, or molecular dynamics simulations. For visualization of the predicted models of the dipeptidyl peptidase via the Foldify platform, visit the following link:

**Figure 6:**
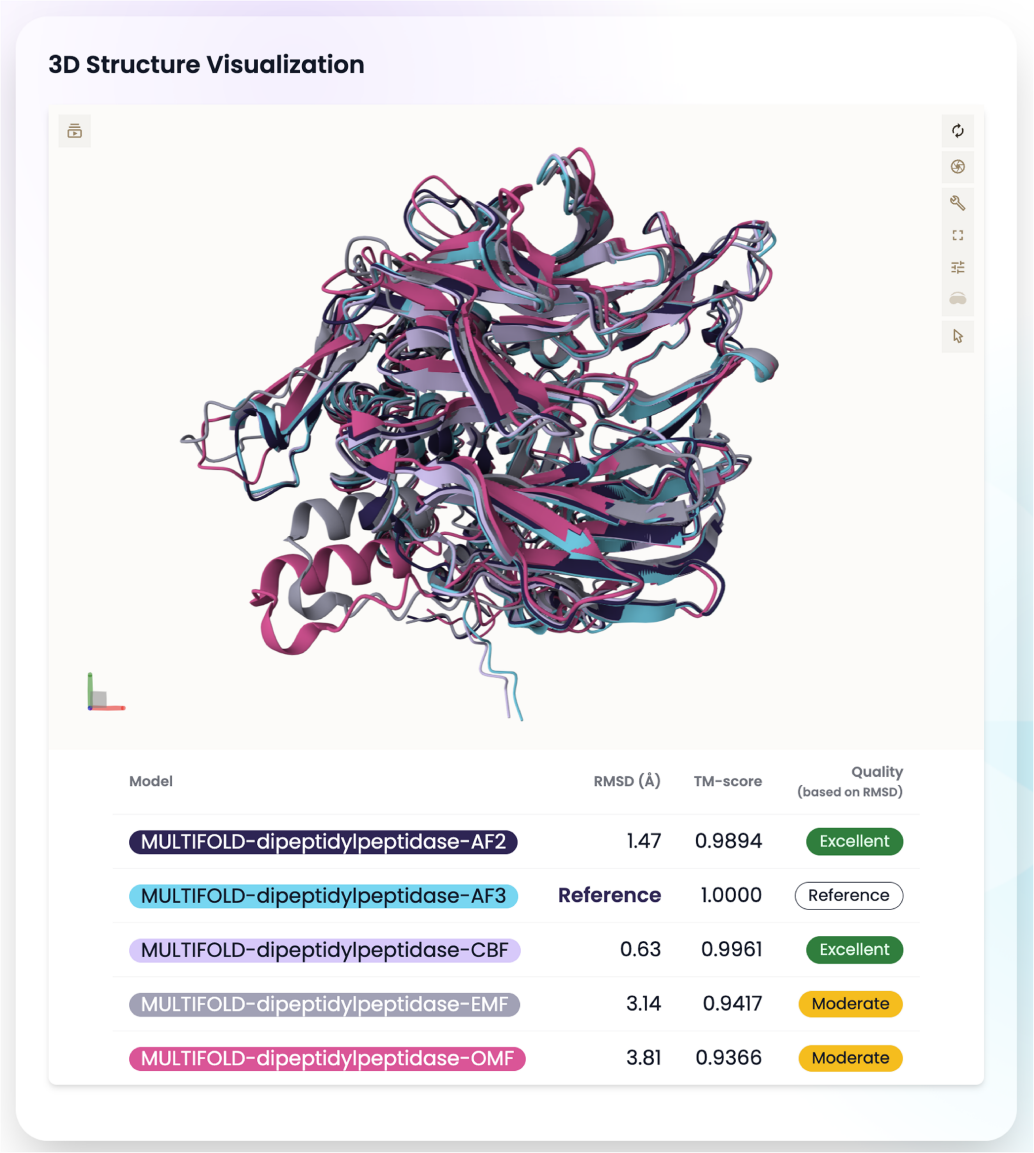
Multimodel comparison of DPP IV N-terminal domain structures predicted by AlphaFold 3, ESMFold, and OmegaFold. The DPP IV protein input sequence was obtained from the AlphaFoldDB entry with UniProt ID A0A8T2CSI4. (Top) Structural alignment of the prediction models rendered in the Mol* Viewer, with AlphaFold 3 shown in dark blue (reference), ESMFold in cyan, and OmegaFold in light purple. (Bottom) Quantitative comparison metrics showing RMSD and TM-scores calculated against the AlphaFold 3 reference. ESMFold achieves closer structural agreement (RMSD: 3.15 Å, TM-score: 0.9415) than OmegaFold (RMSD: 3.82 Å, TM-score: 0.9365).

https://foldify-open.cloud.e-infra.cz/result/multi/MULTIFOLD-dipeptidylpeptidase-AF3_MULTIFOLD-dipeptidylpeptidase-AF2_MULTIFOLD-dipeptidylpeptidase-OMF_MULTIFOLD-dipeptidylpeptidase-EMF_MULTIFOLD-dipeptidylpeptidase-CBF.

## Conclusion

In conclusion, we have developed and now offer a user-friendly web application that facilitates quick and efficient access to computational protein prediction. With Foldify, scientists and researchers can easily submit a protein sequence, generate predictions, visualize results, manage computed data, and compare results from various folding tools. This enables users to focus on data interpretation and analysis rather than on technical details, especially under resource limitations.

For researchers without access to large computational resources, Foldify is available as a ready-to-use web service. For those with high demands for performance and speed, the application is developed to be containerized via Docker images and deployed on local machines, private clouds, or public cloud platforms. Local execution ensures that sensitive data remain within the chosen secure infrastructure.

Additionally, users of the e-INFRA CZ e-infrastructure have access to computational resources that are not limited beyond standard usage policies. Deployed within a scalable Kubernetes environment, Foldify ensures flexible and accessible protein structure prediction that is tailored to diverse user needs.

## Supplementary Material



## Data Availability

Foldify application is freely available at https://foldify-open.cloud.e-infra.cz/ (hosted by the e-INFRA CZ computing cloud), with no login required. The user manual for the application is available at https://docs.cerit.io/en/docs/web-apps/foldify, while the source code is accessible on GitHub under the MIT license at https://github.com/CERIT-SC/foldify-open and is also available in the Supplementary Data. Project name: Foldify; Project home page: https://foldify-open.cloud.e-infra.cz/; Archived version: https://zenodo.org/records/19206976; Operating system(s): Linux/UNIX; Programming language: Python, React; Other requirements: Docker 28.0.1 or higher, Node.js 20.19.1 or higher, npm 10.8.2 or higher, Python 3.10.17, Pip 25.1; License: The Foldify platform source code is released under the MIT License. However, structure predictions generated through the platform rely on third-party models (AlphaFold 3, AlphaFold 2, ColabFold, ESMFold, OmegaFold) that retain their original licensing terms and restrictions. Users are solely responsible for ensuring their use of prediction outputs complies with the respective model licenses, including any noncommercial or attribution requirements. The authors assume no liability for misuse of third-party model outputs accessed via the Foldify platform; Any restrictions to use by nonacademics: None.

## Notes

Video of 3D structure visualization of the proposed use cases: porin, cytochrome P450 and dipeptidyl peptidase IV, predicted by Foldify (MP4)
